# Characteristics of NDM-1-producing *Klebsiella pneumoniae* ST234 and ST1412 isolates spread in a neonatal unit

**DOI:** 10.1186/s12866-018-1334-1

**Published:** 2018-11-14

**Authors:** Xu Huang, Xiangjun Cheng, Pengfei Sun, Chenjie Tang, Fang Ni, Genyan Liu

**Affiliations:** 1 Department of Laboratory Medicine, the First Affiliated Hospital with Nanjing Medical University, Nanjing, 210029 People’s Republic of China; 2 National Key Clinical Department of Laboratory Medicine, Nanjing, 210029 People’s Republic of China

**Keywords:** Multidrug-Resistant, carbapenem-resistant *Klebsiella pneumoniae*, *bla*_NDM-1_, Neonate, Outbreak

## Abstract

**Background:**

The emergence of carbapenem-resistant *Klebsiella pneumoniae* (CR-KP) has become a significant problem worldwide and also being a major threat to children and newborns. Here we report an outbreak of NDM-1-producing *K. pneumoniae* in a neonatal unit.

**Results:**

Six CR-KP strains, isolated from neonates with symptoms of infection, were identified using a VITEK-2 compact system, and the clinical data were retrieved from the electronic case records. In vitro susceptibility testing with broth dilution method showed that all six *K. pneumoniae* isolates were resistant to carbapenems and susceptible to colistin, aminoglycosides, fluoroquinolones and tigecycline. Based on the polymerase chain reaction results, each isolate was found to be *bla*_NDM-1_ gene positive. Clonal relationships were analysed using pulsed-field gel electrophoresis (PFGE) and multilocus sequence typing (MLST) and showed that two different PFGE patterns were formed, which belonged to sequence types ST234 and ST1412. Plasmids carrying *bla*_NDM-1_ were successfully transferred from four of the six isolates to an *Escherichia coli* recipient through conjugative assays. S1-PFGE and Southern blot hybridization showed that four NDM-1-producing *K. pneumoniae* were clonal and carried *bla*_NDM-1_ on the same plasmid. The outbreak was effectively controlled by reducing the potential infection sources. All the patients were successfully treated and recovered after receiving an increased dose of carbapenems. Although the source of this outbreak was not clear, comprehensive measures were carried out and the outbreak was effectively controlled.

**Conclusions:**

ST234 and ST1412 of NDM-1-producing *Klebsiella pneumoniae* are the resistant clone spread in the neonatal unit, comprehensive infection control measures and optimized carbapenem therapy played an important role in controlling this NDM-1-producing *K. pneumoniae* outbreak.

## Background

Carbapenem-resistant Enterobacteriaceae (CRE) has become more and more serious in hospital-acquired multi-drug resistant infections [1]. Patients with CRE infection usually face limited treatment options, prolonged hospitalization, increased economic burden, and even increased mortality*.* Carbapenem-resistant *Klebsiella pneumoniae* (CR-KP) is one of the most popular CRE in hospitals around the world [2]. Carbapenemases are the main mechanism of carbapenem resistance in CRE, including KPC, NDM, IMP, VIM, OXA-48, NMC, GES and SME enzymes [3]. Among them, NDM-1 gene is a novel metallo-beta-lactamase that was first reported in a Swedish patient traveled to New Delhi in 2008 [4]. Since then, Sweden, United Kingdom, Belgium, China, Japan, France, Austria, Germany, Norway, Netherland, Australia and Canada have also reported NDM-1-producing isolates [5].

Since the first *K. pneumonia* isolates harboring *bla*_*NDM-1*_ were reported in Nanchang, China in 2013, they have spread rapidly in China. Even outbreaks of NDM-1 producing *K. pneunoniae* were also reported in Shanghai, Yunnan, Hunan and so on [6–8]. For a long time, CRE infections have mainly come from adult patients. In recent years, the outbreak of CRE infection in newborns has been reported and has attracted more and more attention [6, 9]. For different care model and limited drug selection for neonates, effective infection control and treatment should be further studied [10]. Here, we present an outbreak of NDM-1-producing *K. pneumoniae* in the neonatal ward of a university hospital in China. The molecular characteristics and transmission mechanism of NDM-1-producing *K. pneumoniae* was also studied.

## Methods

### Bacterial isolates and patients

*K. pneumoniae* strains were collected from neonates with symptoms of infection who were admitted to the neonatal ward of a university hospital in Nanjing between June and August of 2016. Identification and in vitro susceptibility tests were carried out and carbapenem-resistance was analyzed with VITEK-2 compact system (bioMérieux, Marcy-l’Étoile, France). The electronic case records including patient demographics, antimicrobial treatment and clinical outcomes were retrieved and reviewed. In order to investigate the source of nosocomial infection, related specimen were collected from incubator surface, head circumference tape, blood machine surface and healthcare worker, isolates named KP7 to KP10 were analyzed.

### Antimicrobial susceptibility testing

The minimum inhibitory concentration (MICs) of imipenem (IPM), meropenem (MEM), ceftazidime (CAZ), cefepime(FEP), aztreonam (ATM), piperacillin /tazobactam (TZP), sulbactam/cefoperazone (SCF), amikacin (AM), piperacillin (PIP), levofloxacin (LE), polymixin B (PB) and tigecycline (TG) were determined using the broth micro dilution method. Two quality control (QC) strains, *Escherichia coli* ATCC 25922 and *K. pneumoniae* ATCC 700603 were used. The results of the antimicrobial susceptibility testing were interpreted as specified by the Clinical and Laboratory Standards Institute (CLSI) [11], except for sulbactam/cefoperazone, colistin and tigecycline, which were interpreted in accordance with the European Committee on Antimicrobial Susceptibility Testing clinical breakpoints (version 6.0) [12].

### Resistant gene detection

PCR was performed as previously described to detect carbapenem resistance genes (*bla*_KPC_, *bla*_IMP_, *bla*_VIM_, *bla*_NDM_ and *bla*_OXA-48_), common extended-spectrum β-lactamase (ESBL) genes (*bla*_CTX-M-1G_, *bla*_CTX-M-2G_, *bla*_CTX-M-8G_, *bla*_CTX-M-9G_, *bla*_TEM_, *bla*_OXA-1_ and *bla*_SHV_), plasmid mediated quinolone resistance (PMQR) genes (qnrA, qnrB, qnrS, and aac-(6′)-Ib-cr) and 16S rRNA methylase genes (*armA*, *rmtB*) in all strains [13–15]. PCR amplicons were sequenced, and the sequencing results were compared to the database at the National Center for Biotechnology Information (NCBI) (https://www.ncbi.nlm.nih.gov/genbank/) using BLAST searches.

### Molecular typing

NDM-1-producing strains were genotyped using PFGE and MLST [16]. The allelic profiles and sequence types (ST) were available in the MLST database (http://bigsdb.pasteur.fr/klebsiella/klebsiella.html). Clonal relationships were analysed using PFGE and bacterial DNA was digested with XbaI endonuclease (TaKaRa, Dalian, China) as previously described [7]. Salmonella enterica serotype H9812 was used as a marker. The PFGE patterns were compared using BioNumerics software (Applied Maths, Kortrijk, Belgium), and a phylogenetic tree was built for cluster analysis. Clusters were defined as DNA patterns sharing > 90% similarity.

### Conjugation experiments

Conjugative assays were performed using sodium azide-resistant *E. coli J53* as the recipient strain (donated by professor Wenen Liu, Central South University) [17]. Transconjugants were selected on MacConkey agar plates containing 150 mg/L sodium azide (Sigma Aldrich, St Louis, MO) and 30 mg/L ceftazidime (Sigma-Aldrich) for 24 h at 35 °C. Presence of the *bla*_NDM-1_ gene in the transconjugants was confirmed by PCR analysis, and their antimicrobial susceptibilities were determined as well.

### Plasmid analysis

S1-PFGE and Southern blotting were performed to analyse the size of the NDM-1-carrying plasmids in the *K. pneumoniae* strains as previously described and the results were analyzed according to the criteria proposed by Tenover et al. [18, 19] Plasmid DNA of the isolates embedded in agarose gel plugs were digested with S1 nuclease and separated by PFGE. Plasmids obtained by PFGE were then transferred to a positively charged nylon membrane. The membrane was hybridized with digoxigenin-labelled *bla*_NDM-1_-specific probes and the signals were detected using an NBT/BCIP colour detection kit (Roche Applied Sciences, Penzberg, Germany).

## Results

### Clinical characteristics of the *K. pneumoniae* isolates

The clinical profiles of the six NDM-1-positive *K. pneumoniae* isolates (KP1 to KP6) are shown in Table 1, including the patient demographics, the date of specimen isolation, specimen source and clinical diagnosis, antimicrobial treatment and clinical outcomes. The six NDM-1-positive *K. pneumoniae* isolates were obtained from blood (*n* = 5) and sputum (*n* = 1). Five patients had sepsis, and one had neonatal respiratory distress syndrome. No infants died from their infections after effective treatment. The newborns experienced long hospitalization periods (mean, 62 days), and treatment with increased doses of carbapenem showed good prognoses.

**Table 1:** Clinical profiles of the six NDM-1-positive *K. pneumoniae* isolates

Case	Sex	Pregnancy duration (wk)	Sample type	Type of infections	Antimicrobial therapy	PFGE type	ST type	NDM-coding plasmids (Kb)
1	F	32	Blood	Neonatal sepsis, neonatal respiratory distress syndrome	meropenem^a^	A	234	none
2	F	31	Blood	Neonatal sepsis, neonatal respiratory distress syndrome	meropenem	A	234	none
3	M	26	Sputum	Neonatal respiratory distress syndrome	imipenem^b^	B	1412	50
4	M	30	Blood	Neonatal sepsis, neonatal respiratory distress syndrome	meropenem	B	1412	50
5	M	35	Blood	Neonatal sepsis, neonatal pneumonia	meropenem	B	1412	50
6	M	29	Blood	Neonatal sepsis	meropenem	B	1412	50

^a^meropenem 0.5 g IVD,qd; ^b^ imipenem 1.0 IVD qd;

### Antibiotic susceptibility testing and characteristics of drug-resistant genes

All six clinical isolates were resistant to imipenem, meropenem, ceftazidime, cefepime, piperacillin, piperacillin /tazobactam, sulbactam/cefoperazone and aztreonam (except for KP6, MIC = 0.125 mg/liter) but remained susceptible to amikacin, levofloxacin, polymixin B and tigecycline. The transconjugants had similar susceptibility results. Among the six NDM-1-positive isolates, 67% (4/6) co-harboured *bla*_SHV-148_ and 33% (2/6) co-harboured *bla*_CTX-M-14_ *bla*_SHV-27_ and qnrB4. Other resistance genes were not detected. Results of antibiotic susceptibility testing, drug-resistant genes analysis of clinical isolates and respective transconjugants are shown in Table 2.

**Table 2:** Antimicrobial susceptibilities of the six NDM-1-positive *K. pneumoniae* isolates

Isolate No.	Resistance mechanisms	MIC(mg/liter) of^a^											
		SCF	IPM	CAZ	MEM	TZP	PB	FEP	AM	PIP	LE	TG	ATM
KP1	NDM-1, CTX-M-14, SHV-27,qnrB4	> 128	64	> 128	64	> 128	0.5	64	0.125	> 128	< 0.125	0.125	4
KP2	NDM-1, CTX-M-14, SHV-27,qnrB4	> 128	64	> 128	64	> 128	0.5	64	0.125	> 128	< 0.125	< 0.125	4
KP3	NDM-1, SHV-148	> 128	32	> 128	> 128	> 128	2	> 128	0.25	> 128	< 0.125	0.25	4
KP4	NDM-1, SHV-148	> 128	32	> 128	> 128	> 128	1	> 128	0.25	> 128	< 0.125	< 0.125	4
KP5	NDM-1, SHV-148	> 128	32	> 128	> 128	> 128	1	64	1	> 128	< 0.125	< 0.125	8
KP6	NDM-1, SHV-148	> 128	32	> 128	32	> 128	1	32	1	> 128	< 0.125	< 0.125	0.125
J53–3	NDM-1	> 128	16	> 128	32	> 128	2	128	8	> 128	< 0.125	< 0.125	0.25
J53–4	NDM-1	> 128	32	> 128	32	> 128	2	64	4	> 128	< 0.125	< 0.125	0.25
J53–5	NDM-1	> 128	8	> 128	8	> 128	2	32	2	> 128	< 0.125	< 0.125	< 0.125
J53–6	NDM-1	> 128	8	> 128	8	> 128	2	32	4	> 128	< 0.125	< 0.125	0.25

^a^*SCF*, sulbactam/cefoperazone; *IPM*, imipenem; *CAZ*, ceftazidime; *MEM*, meropenem; *TZP*, piperacillin /tazobactam; *PB*, polymixin B; *FEP*, cefepime; *AM*, amikacin; *PIP*, piperacillin; *LE*, levofloxacin; *TG*, tigecycline; *ATM*, aztreonam

### Molecular epidemiology

**Fig. 1:**
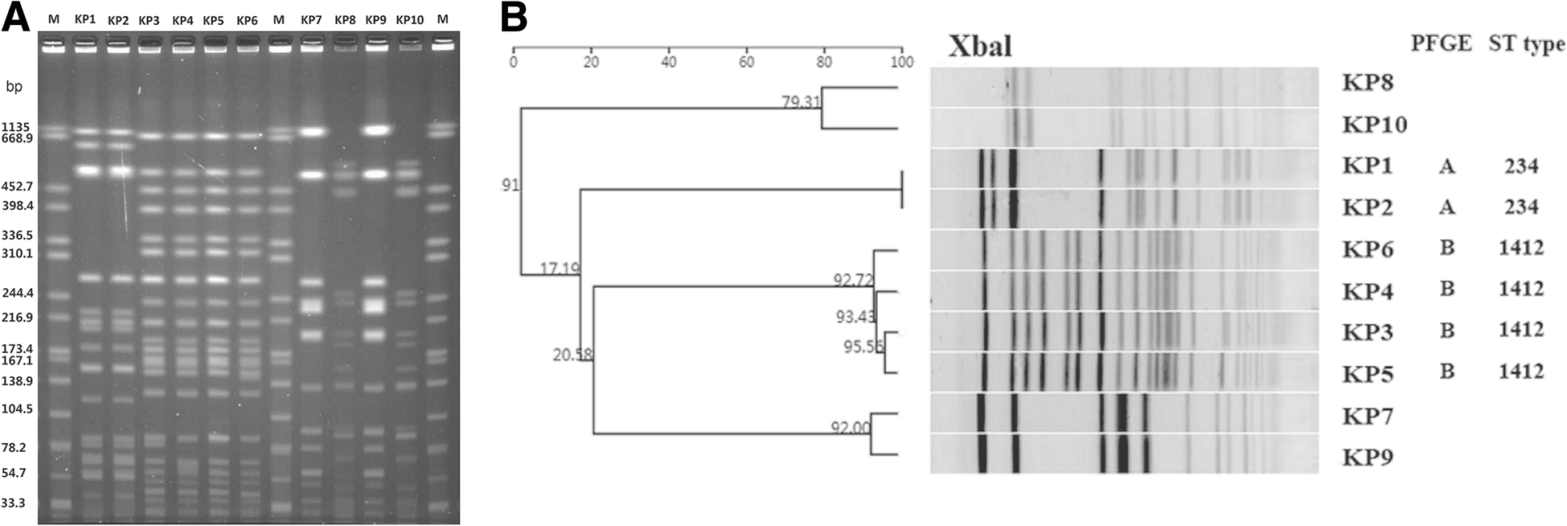
Pulsed-field gel electrophoresis analysis of isolated NDM-1- producing *K. pneumoniae* strains. A**,** Pulsed-field gel electrophoresis fingerprint of the isolates; B, Molecular epidemiology investigation of the isolates; KP1 to KP6: patients; KP7: incubator surface; KP 8: head circumference tape; KP 9: blood machine surface; KP 10: healthcare worker; lane M: marker (Salmonella H9812)

Two distinct PFGE patterns were obtained from the *XbaI* DNA digests among the six CR-KP isolates: type A (*n* = 2) and type B (*n* = 4). Carbapenem-susceptible *K. pneumoniae* isolated from the incubator surface, head circumference tape, blood machine surface, and medical workers showed no homology with the six CR-KP isolates (Fig. 1a). Two distinct sequence types were obtained from the six isolates:KP1 together with KP2 are ST234 and KP3, KP4, KP5 and KP6 are ST1412. Comparison of these results showed that all PFGE type A isolates corresponded to ST234, and the type B isolates corresponded to ST1412(Fig. 1b).

#### Transfer of carbapenemase resistance

Transconjugants were obtained in the conjugation experiments, with a success rate of 67%. In the conjugation experiments, the *bla*_NDM-1_ genes were transferred successfully from the donor *K. pneumoniae* isolates (except KP1 and KP2 isolates) to the recipient *E. coli J53* via plasmids. Antimicrobial susceptibility detection showed that all transconjugants displayed carbapenem resistance (Table 2). No other resistance genes were detected. The conjugation experiments were conducted multiple times with KP1 and KP2, but the results were always negative. All transconjugants revealed similar antimicrobial susceptibility as that of the donor strains.

#### Plasmid analysis

**Fig. 2:**
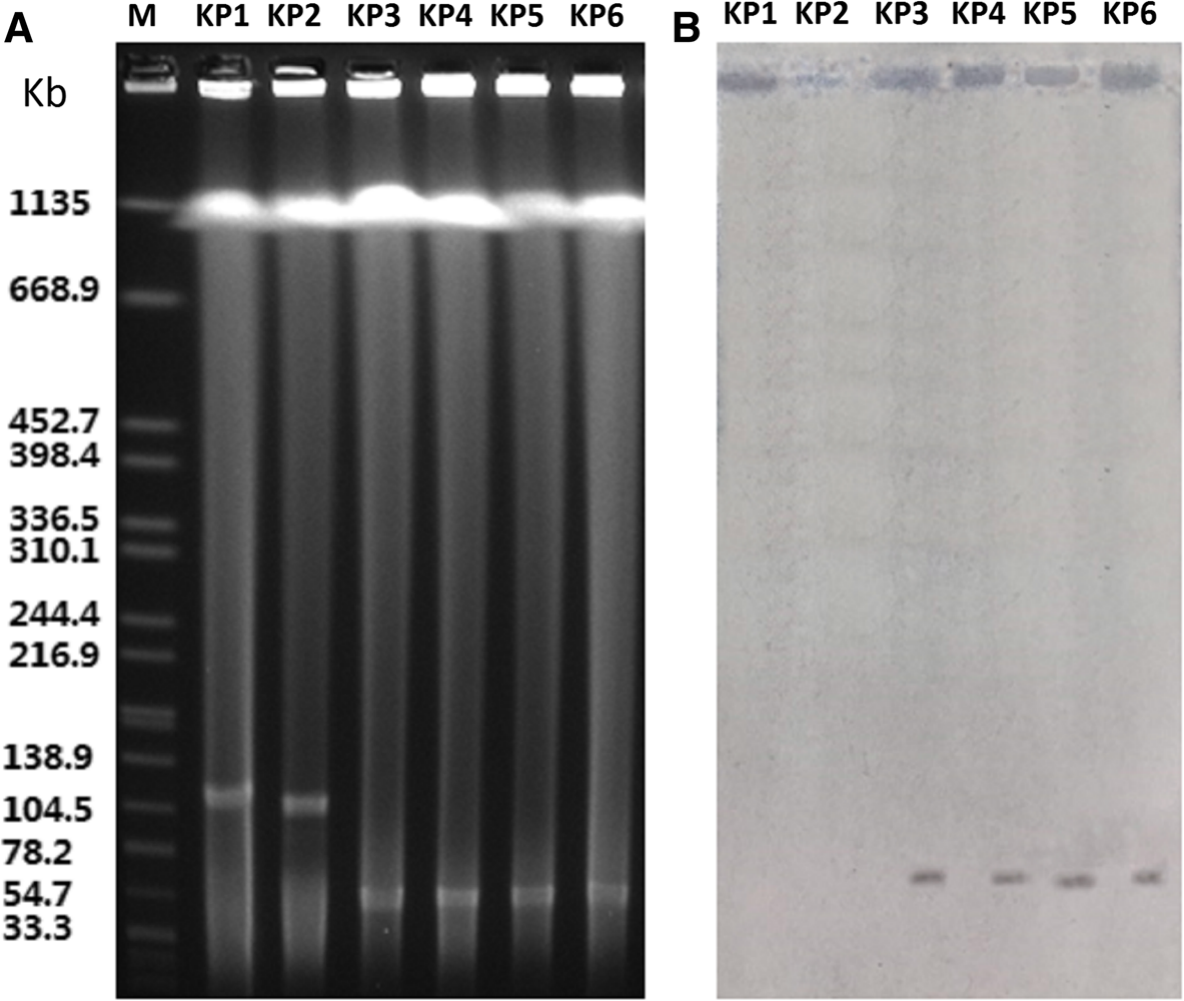
*blaNDM-1*-carrying plasmid analysis. A S1 nuclease pulsed-field gel electrophoresis (PFGE); B Southern hybridization using a *blaNDM-1*-specific probe; Lane M: marker (Salmonella H9812); KP1 to KP6: clinical isolates

S1-PFGE and Southern blot hybridization analysis indicated that the *bla*_NDM-1_ genes were transferred via the same plasmids (with approximate size 50 kb), except for in the KP1 and KP2 strains (Fig. 2b). Although the KP1 and KP 2 strains carried plasmid DNA (with approximate size 110 kb) (Fig. 2a), they did not seem to transmit the *bla*_NDM-1_ gene via these plasmids (Fig. 2b). Additionally, conjugation experiments and Southern blotting hybridization showed that the *bla*_NDM-1_ gene probably falls within the chromosome of KP1 and KP2 isolates.

## Discussion

Infectious diseases caused by NDM-1-producing *Enterobacteriaceae* are known to be associated with high morbidity and mortality worldwide. This is a great challenge for paediatricians due to the transferability of the gene and the poor prognosis in children. Of the carbapenem resistance genes found in carbapenem-resistant *Enterobacteriaceae* in children, NDM genes (including coding genes of NDM-1 and NDM-6) are more common than KPC and VIM [10]. Compared to adults, children are more limited in antibiotic use. Although these isolates were all susceptible to tigecycline and colistin in vitro, the paediatrician had not used either of these treatments because of their potential side effects in children. Aminoglycosides and fluoroquinolones are also not used for children due to their nephrotoxicity and ototoxicity [6]. After careful consideration, the paediatrician in this report chose to use an increased dose of carbapenems (meropenem 0.5 g IVD qd; imipenem 1.0 g IVD qd), and no side effects were reported during the antimicrobial therapy. Fortunately, all of the neonates in this report had positive prognoses. However, the proposed doses of meropenem and imipenem for neonates who are greater than 28-days-old are 120 mg/kg/day and 60–100 mg/kg/day, respectively, according to the Sanford Guide to Antimicrobial Therapy. In the present study, the doses used are more than 4 times the amount suggested by the guide. Thus, a recent study showed that monotherapy for treatment of CRE infection in adults was associated with higher mortality than that of combination therapy, and limited data were available for children [10, 20, 21]. Although we had a good prognosis this time, further dosage optimization of carbapenem and medication for antimicrobial therapy for NDM-1-producing *Enterobacteriaceae* should be investigated.

In our study, PFGE analysis showed two clusters of the NDM-1-producing strains that corresponded to ST234 and ST1412. Among these, none were found to be clonally related to the carbapenem-susceptible *K. pneumoniae* isolated from the surface of incubators, blood machines, neonatal head circumference tapes and medical workers. Regarding the epidemiology of this spread, 2 isolates (5, 6) were identified in June, and 4 isolates (1, 2, 3, and 4) were identified in August. This means that the ST1412 isolates remained in the neonatal ward for 2 months and reappeared in August, along with a new strain. It is unfortunate that although we have not found the source of this outbreak, we cannot deny that the hospital environment was likely the source of these infections. After all, the incubator water and the sharing of breast milk has been reported to be ‘hotspots’ for bacterial transmission in neonatal wards [7, 22]. When we first confirmed the outbreak, our hospital control team took active measures to sterilize the source of infection in the environment and restrict the admission of newborns to the unit. Finally, the outbreak event was successfully controlled and all infected patients recovered and were discharged. NDM-1-producing *K. pneumonia* have been reported as belonging to various types of MLST: ST11, ST14, ST17, ST25, ST37, ST76, ST105, ST147, ST149, ST231, ST340, and ST1043 [6, 7, 23]. Our data indicate that NDM-1-producing *K. pneumoniae* strains belong to two independent types, ST234 and ST1412, which differ from most types reported previously, particularly for the never reported NDM-1 producing ST234 strains, which have only been reported as KPC-producing *K. pneumonia* [24].

The plasmid analysis in this study showed that the *bla*_NDM-1_ genes transferred using the same plasmids (with approximate size 50 kb), except for the KP1 and KP2 strains. We analysed the genomic DNA of four transconjugants using PCR, and the results showed that only *bla*_NDM-1_ was present. This result indicates that *bla*_SHV-148_ was not on the same plasmid as *bla*_NDM-1_. For the KP1 and KP2 strains, we speculated that the *bla*_NDM-1_ gene may be in the chromosome or may be a high-molecular-weight non-conjugative plasmid that was not detected by the methodology used here. Further studies should be investigate this gene. The source of the NDM-1 has not been determined. It has been studied in previous *bla*_NDM_ variants from sewage water in hospitals, which serves as an important source of the spread of antibiotic resistance genes [25]. Guidance from the European Centre for Disease Prevention and Control suggested that intervention measures should be taken on antimicrobial stewardship, clean hospital settings, equipment reprocessing, staff education and microbiological capacity to minimize risks of spread of CRE [26]. All we can do is strengthen the unity of the clinical departments to prevent further infection.

## Conclusions

ST234 and ST1412 of NDM-1-producing *Klebsiella pneumoniae* are the resistant clone spread in the neonatal unit, comprehensive infection control measures and optimized carbapenem therapy played an important role in controlling this NDM-1-producing *K. pneumoniae* outbreak.

## Abbreviations

CLSI: The Clinical and Laboratory Standards Institute
CRE: Carbapenem resistant Enterobacteriaceae
CRKP: Carbapenem-resistant *Klebsiella pneumoniae*
ESBL: Extended-spectrum β-lactamase
MIC: Minimum inhibitory concentration
MLST: Multilocus sequence typing
PCR: Polymerase chain reaction
PFGE: Pulsed-field gel electrophoresis
